# New all oral therapy for chronic hepatitis C virus (HCV): a novel long-term cost comparison

**DOI:** 10.1186/s12962-015-0043-y

**Published:** 2015-10-06

**Authors:** Jennifer M. Poonsapaya, Michael Einodshofer, Heather S. Kirkham, Pheophilus Glover, Janeen DuChane

**Affiliations:** Walgreen Co., 1415 Lake Cook Road, MS #L411, Deerfield, IL 60015 USA; Walgreen Co., 500 Noblestown Road, Ste. 200, Carnegie, PA 15106 USA; Baxalta US Inc., 1200 Lakeside Drive, Bannockburn, IL 60015 USA

**Keywords:** Hepatitis c virus, Hepatitis c prevalence, Cost-savings analysis, Economic modeling, Managed care, Hepatitis c treatment, Interferon, Antiviral agents, All oral therapy

## Abstract

**Background:**

In the US, the prevalence of hepatitis C virus (HCV) has surpassed the prevalence of human immunodeficiency virus (HIV), with about 3.3 million people chronically infected with the disease. Given the aging of the Baby Boomer generation and the subsequent implementation of age-based screening recommendations, HCV diagnoses are expected to increase. Utilization of anti-viral pharmacotherapy is also expected to increase as more effective and tolerable all-oral therapies for HCV become available in the United States. This research allows payors to assess the disease burden and treatment impact of HCV in their member group.

**Methods:**

A set of three integrated economic models was developed to estimate the disease and cost burden of HCV based on existing literature, wholesale acquisition costs, industry standards, and actuarial judgment. Model 1 estimates the HCV antibody prevalence of HCV in a payer’s member group based on population size and the age, sex, and region distribution of the members. Model 2 predicts the number of uncured chronic HCV members who represent the future treatment and medical cost burden for the payer over the next 14 years. Model 3 contrasts the pharmacy, medical, and overall costs for treatment and medical care over 14 years for three therapeutic scenarios: interferon-based standard of care (SOC), all oral therapy, and natural course of disease progression, while accounting for the frequency of HCV genotype within the member population.

**Results:**

In a payer population of 100,000 members with an age, sex, and region distribution matching the United States, the seroprevalence of HCV was estimated to be 1.26 %. Combined pharmacy and medical costs for uncured chronic HCV positive members was least expensive for all oral therapy. The per patient with HCV cost savings for all oral therapy compared to SOC were about $3000 per year over 14 years. In a sensitivity analysis, the 12-week all oral therapy for genotype 1 provided overall cost savings vs. a 24-week interferon-based SOC regimen until all oral therapy costs exceeded $99,000.

**Conclusions:**

In most modeled scenarios, the all-oral therapeutic scenario was less costly than SOC, even in sensitivity analyses.

## Background/rationale

### Descriptive epidemiology

Hepatitis C virus (HCV) is the most prevalent blood borne pathogen in the United States, with about three million people chronically infected in the non-institutionalized population [1]. There are approximately 17,000 new infections and 15,000 deaths annually due to the disease [2]. As of 2013, 75 % of people infected with HCV were between 48 and 68 years of age [3]. Men and African Americans are also more likely to test positive for HCV [4]. The main risk factor for acquiring HCV is injection drug use, accounting for about 50 % of infections [3]. Another risk factor is a history of receiving a blood transfusion or organ transplant before the year 1992 because the blood supply was not screened for HCV antibodies during that time. Other risk factors include: receipt of a blood clotting factor prior to 1987, long-term hemodialysis, presence of a known exposure (i.e., needle stick from HCV infected person or organ transplant from known HCV carrier), having HIV, or being born to a mother with HCV [2].

Hepatitis C infections have two stages: acute and chronic. Approximately 75–85 % of acute infections progress to chronic disease over time in the absence of diagnosis and treatment [2]. There are three predominant genotypes of HCV in the United States. The most prevalent is genotype 1, accounting for 73 % of infections, followed by genotypes 2 and 3 accounting for the vast majority of the remaining 27 % of infections [5]. Most people do not know that they are infected in either the acute or chronic stage of the disease. Studies suggest that only about one of every four persons infected with chronic HCV has received a diagnosis; this number improves to one out of every two in populations with access to care [6, 7]. Given the prevalence of undiagnosed disease, many learn of their disease only when symptoms of chronic infection appear, generally about 20–30 years after infection [4]. If the infection is allowed to progress naturally, approximately 27 % of those with chronic HCV infection will develop cirrhosis [8]. Among those with cirrhosis, about 25 % will progress to end-stage liver disease or hepatocellular carcinoma [4]. As such, HCV is the leading cause of liver transplantation in the United States [2].

The number of people diagnosed with chronic HCV infection will rise rapidly in the United States in the coming years [9]. This dramatic increase is due a couple of factors. First, a large proportion of those infected, namely the Baby Boomer generation (born between 1945 and 1965), is nearing the end of the latency period and will begin to show symptoms of cirrhosis prompting them to seek care. As such, cirrhosis prevalence is anticipated to reach about 1 million people in 2020 [3]. Second, the advent of age-based screening recommendations for the Baby Boomer group will lead to an increase in diagnoses among asymptomatic people—the US Preventive Services Task Force has recommended that all individuals born during the period 1945–1965 receive a one-time screening for HCV [3].

### Treatment

The goal of HCV therapy is to prevent morbidity and mortality resulting from the virus [10]. According to the CDC, the rate of spontaneous viral clearance is relatively low (15–25 %) [2]; thus, in order for a chronically infected person to clear the virus from their body anti-viral pharmacotherapy is generally required. Viral eradication is determined clinically via a measure called sustained virologic response (SVR), which indicates current laboratory technology cannot detect any virus in the bloodstream for a defined period of time, generally 12 weeks, after concluding pharmacotherapy [10, 11]. The treatment of chronic HCV depends upon the genotype. Until November 2013, the standard of care (SOC) therapy for HCV was interferon-based. For genotypes 2 and 3, the regimen consisted of ribavirin plus pegylated interferon for 24 weeks, leading to a sustained virologic response rate of 81 % [12, 13]. For those with genotype 1, accounting for the majority of HCV infections, the SOC up until November 2013 was a triple therapy regimen of ribavirin, pegylated interferon, and a protease inhibitor (boceprevir or telaprevir) recommended for up to 48 weeks [14, 15]. Sustained virologic response for those with genotype 1 on triple therapy ranged from 66 to 75 % depending on the protease inhibitor used [14].

The interferon-based SOC therapy across genotypes posed several challenges. First, side effects from treatment were common. Up to 14 % of patients discontinued therapy because of adverse events [16, 17]. The most common side effect reported was flu-like symptoms, often occurring for 12 months and in over half of patients. Other common side effects included depression, irritability, and inability to sleep, each of which occur in about one quarter of patients [10]. Anemia and skin rash are also possible side effects from ribavirin [10]. Second, administration was complicated by the fact that interferon was administered via self-injection.

The shortfalls of the interferon-based SOC therapy included (a) sub-optimal SVR rates amongst all genotypes, (b) side effects of therapy, and (c) the complexity of therapy administration. As such, adherence and subsequent cure rates were suboptimal [18]. This coupled with the fact that the number of people with chronic infection becoming aware of their disease is increasing [19] means that opportunities exist for new and better HCV therapies to enter the marketplace.

Several drug companies have responded and are developing HCV therapies to overcome the shortfalls of past SOC treatment. All oral regimens currently in the drug pipeline appear to have several advantages, compared to SOC, including much higher SVR rates (typically 90 % and higher) and fewer side effects due to the removal of interferon, and sometimes ribavirin, replacing them with more targeted agents [20, 21]. The advantages of all oral therapy have prompted some physicians to refrain from treating patients with SOC regimens in anticipation of better all oral therapies on the horizon [22–25].

Recently, sofosbuvir was approved by the US Food and Drug Administration (FDA) for use with ribavirin as an all-oral regimen to treat genotype 2 and 3 disease, respectively [21]. At the time of this research, however the combination of sofosbuvir and ledipasvir as an all oral regimen for the treatment of genotype 1 disease was still under priority review by the FDA [26].

Of note, Walgreen Co. makes no recommendation or endorsement of any drug or treatment regimen. Information for all approved drugs listed in this publication, including any applicable boxed warning, is available at http://dailymed.nlm.nih.gov/dailymed.

### Study rationale

Due to the anticipated increase in HCV diagnoses in the coming years and the new all-oral therapies on the horizon, payers need to be informed of the potential impact of HCV in the member population they serve. We hypothesized that the use of all oral therapy to treat patients with chronic HCV would be less expensive over time than treating those patients with SOC or allowing them to proceed through the natural course of the disease without any treatment.

## Methods

We developed a set of three integrated economic models to assist payers with projecting the disease and cost burden of treating uncured chronic HCV infection in a member population. The integrated economic models were based upon assumptions derived from peer-reviewed literature, industry standards, wholesale acquisition costs, and actuarial judgment. The models also underwent a thorough review by an outside actuarial firm that validated our methodology. Below we describe the assumptions as well as the inputs and outputs of each model.

### Model 1

#### Input: population and demographics

**Fig. 1:**
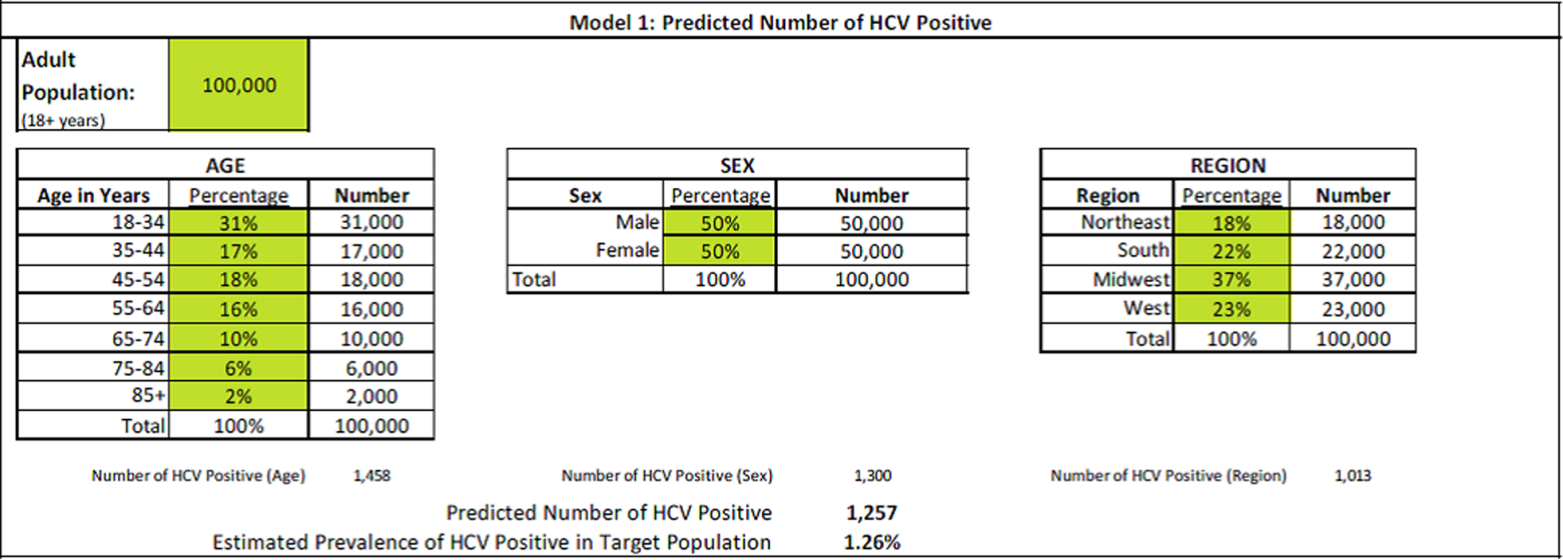
Model 1 inputs and outputs

In Model 1, the user is given the ability to customize the size of the population and the distribution of the population across age, sex, and region categories because HCV prevalence has been shown to vary by these demographic characteristics [27, 28]. The default member population was set to 100,000 as this number is generally used to express the HCV prevalence at the population level [28]. The default age distribution for the population was based on the age distribution for the United States from the 2012 American Community Survey [29]. As the population for this project is limited to persons aged 18 years and older, which comprises 76.5 % of the total US population, we divided the percentage of the population within each age group by 76.5, so that the sum of the age group percentages totaled 100 %. The age groupings and corresponding percentages are shown in Fig. 1. The sex distribution default was set to 50 % male and 50 % female. The region distribution was based on the census region level from the 2010 Census [30]. The default regional distribution for the population can also be found in Fig. 1. Of note, all numbers and percentages shown in green boxes may be modified by the user (Fig. 1).

#### Output: HCV prevalence

HCV antibody prevalence was calculated based on the average of the age, sex, and region-specific prevalence estimates. The source of the data for both the age- and sex-specific HCV prevalence estimates was the National Health and Nutrition Examination Survey (NHANES) 2007–2008 data [6, 27]. HCV prevalence for males and females was 1.6 and 1.0 %, respectively. The age-based prevalence estimates were projected out 5 years to estimate the HCV prevalence based on the age groups 5 years later (2012–2013). HCV prevalence by age category (in years) was as follows: 18–34: 0.20 %, 35–44: 1.40 %, 45–54: 3.60 %, 55–64: 2.40 %, 65–74: 0.70 %, 75–84: 0.70 %, 85 and older: 0.70 %.

Region-specific prevalence information was not readily available via published literature; therefore, we extrapolated HCV prevalence based on data available from the reported acute HCV cases by state (including the District of Columbia). The numerator was derived by taking the average number of reported acute cases within a state over the three most recent years, 2009–2011, and multiplying it by 13.4 [28] to estimate the actual number of acute HCV cases. Then, we took the actual number of acute cases and divided it by the ratio of acute to total cases (i.e. acute + chronic) of HCV in the United States to estimate the total number of HCV antibody positive in each state. The denominator was based on decennial census data from 2010 for each state and the District of Columbia [31, 32].

$$ {\text{Numerator = }}\frac{{\left( {\frac{\text{a1 + a2 + a3}}{ 3}} \right)}}{{\frac{\text{B}}{\text{B + C}}}} $$

a1 = number of reported acute cases of HCV for the state in 2009

a2 = number of reported acute cases of HCV for the state in 2010

a3 = number of reported acute cases of HCV for the state in 2011

$$ \left( {\frac{\text{b1 + b2 + b3}}{ 3}} \right) $$

b1 = number of reported acute cases of HCV for the US in 2009

b2 = number of reported acute cases of HCV for the US in 2010

b3 = number of reported acute cases of HCV for the US in 2011

C = average estimated number of chronic cases of HCV in the US.

To create region-level prevalence estimates, we aggregated the state-level numerator and denominator data to the US census region level. The reported number of acute HCV cases was unavailable for Alaska, Arizona, Delaware, Hawaii, Mississippi, New Hampshire, Rhode Island, and South Dakota. Thus, numerator data for those states was extrapolated based on that region’s prevalence estimate and the respective state’s population. Region estimates were then recalculated to include the states with extrapolated data. We estimated HCV prevalence to be the following: 1.20 % in the Northeast, 1.48 % in the South, 0.74 % in the Midwest, and 0.86 % in the West.

### Model 2

**Fig. 2:**
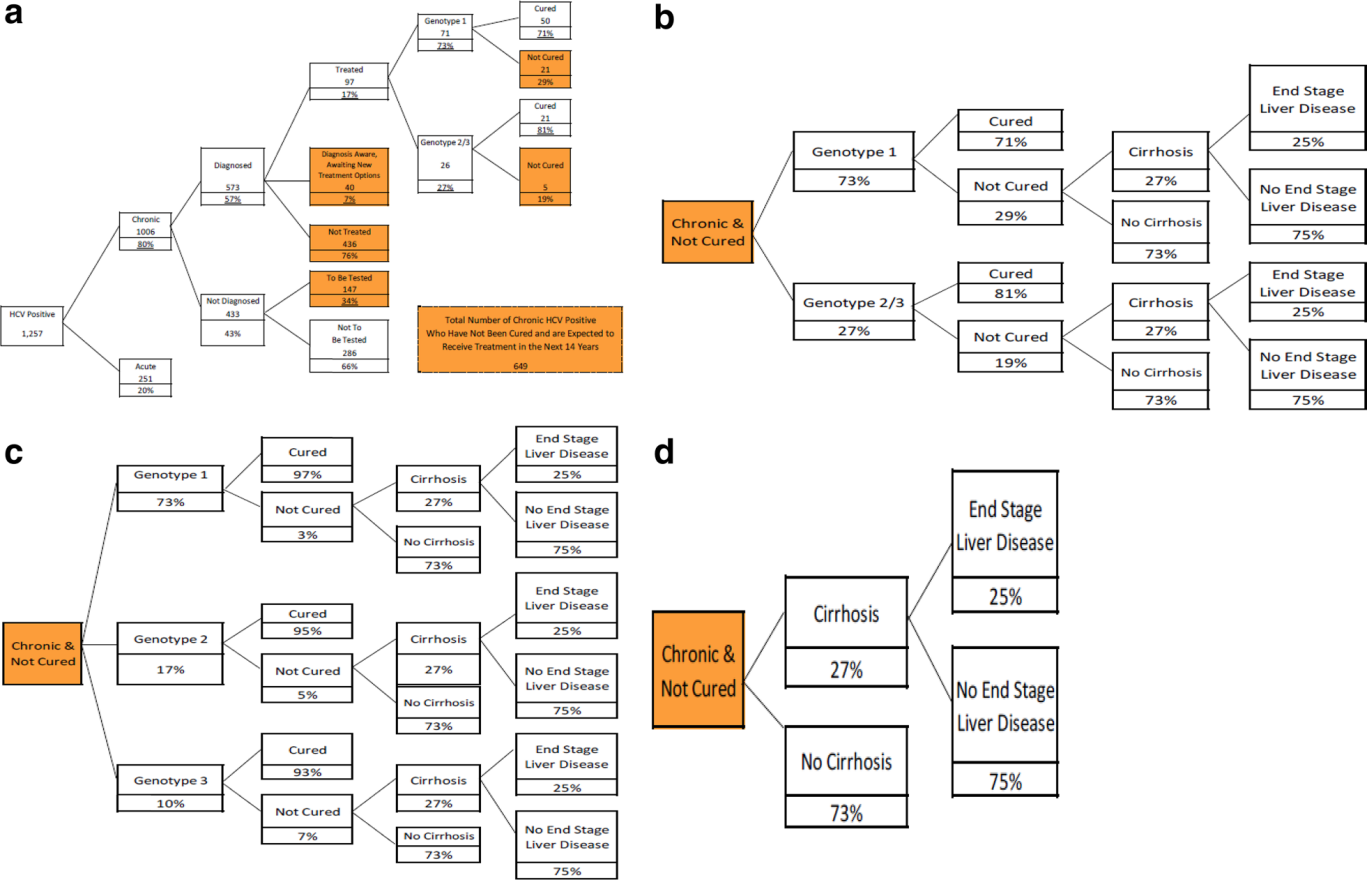
**a** Model 2 flow diagram. *Shaded areas* estimate the source of 649 individuals from a commercial benefits program comprising 100,000 members who would be expected to receive treatment for HCV within 14 years. This group would be responsible for the treatment and medical burden to the payer over the subsequent 14 years. **b** Standard of Care: cure rates and therapeutic endpoint probabilities. “Standard of Care” = interferon-based treatments used prior to November, 2013. **c** All Oral Therapy: cure rates and therapeutic endpoint probabilities. “All Oral Therapy” = various combinations of sofosbuvir, ledipasvir and ribavirin. **d** Natural course of disease progression: therapeutic endpoint probabilities. “Natural Course of Disease Progression” = no treatment

The output from Model 1 (i.e., the number of people who were expected to be HCV antibody positive in the target population) provides the input for Model 2, which is a classification tree used to estimate the number of members with uncured chronic HCV. For Model 2, we estimated that 80 % of those infected with HCV would have chronic infection [2], some of whom would not be diagnosed yet (Fig. 2). Among those with chronic infection, we estimated the number of uncured using the following scenarios: (a) those with undiagnosed chronic HCV who will be tested and diagnosed in the future, (b) those with chronic HCV who were diagnosed but not treated, and (c) those with chronic HCV who were diagnosed and treated but treatment was not successful (i.e., treatment failure; see Fig. 2). Support for key assumptions in Model 2 are as follows:

- Spradling and colleagues estimated that approximately 43 % of people with HCV and access to care have not been diagnosed [6]. In order to receive treatment, a diagnosis must be made. Therefore, amongst those chronically infected and undiagnosed, we assumed that only those people approximating the baby boomer generation (aged 45–64 in 2013) from Model 1, or 34 % of those chronic and not diagnosed with access to care, would be tested over the next 14 years. We assumed all those in the Baby Boomer generation who were chronic and not diagnosed would be tested per the US Preventive Services Task Force (USPSTF) recommendation that all people in the Baby Boomer generation be tested for HCV, due to the high prevalence of infection in this group [33].
- For the 57 % of people with chronic HCV whom we predicted would be diagnosed [6], it is estimated that about three quarters would be untreated [34], leaving 24 % who would have historically received treatment. However, with the onset of physicians refraining from treating patients until new therapies are available, we estimated that 30 % of the diagnosed who historically would have been treated (i.e., 30 % of 24 % = 7 %) are now awaiting new therapy [23]. After taking into account those awaiting new therapy, we estimated that only 17 % (24 % minus 7 %) of the diagnosed had received treatment.
- Among those who have received treatment, we estimated that 73 % were genotype 1, and, for simplification purposes, the remaining 27 % were genotype 2 or 3 [5]. Those with genotype 1 disease who were treated we assigned a treatment naïve cure rate of 71 % (combined cure rate assuming half were on telaprevir-based regimen and half were on a boceprevir-based regimen) [14]. For those with genotype 2 or 3 disease, we estimated a cure rate of 81 % by averaging the cure rates from various studies [12, 13].

Taking into account the three scenarios above, approximately 52 % of the target population who was HCV antibody positive was considered to have chronic HCV that has not yet been cured; this reflects the future disease burden for the payer over the next 14 years (Fig. 2).

### Model 3

The output from Model 2 served as the input for Model 3, which predicted the cost of pharmacy and subsequent medical expenses over 14 years following treatment for three therapeutic scenarios: (a) SOC treatment, (b) all oral treatment, and (c) natural course of disease progression. The natural course of disease progression was included for benchmarking purposes only as the lack of treatment would be considered unethical. Below we outline the assumptions incorporated into Model 3.

#### Cure rates

Therapy cure rates were based upon published literature for treatment naïve individuals since 96 % of the people from Model 2 with uncured chronic HCV would not have received prior treatment (Model 2). Table 1 outlines the cure rate we assigned by genotype within each treatment scenario.

**Table 1:** Sensitivity analysis by input for each model

Variable	Baseline scenario	Input range	Output range (baseline: 1257)	Input range References/comments
Model 1				
% Age in years		100 % 18–34 years–100 % 45–54 years	838–1971	Theoretical range
% Male	50 %	0–100 %	1157–1357	Theoretical range
% Region		100 % Midwest–100 % South	1166–1413	Theoretical range

Variable	Baseline scenario (%)	Input range (%)	Output range (baseline: 649)	Input range References/comments
Model 2				
Proportion diagnosis aware, not yet treated	30	20–40	639–659	[25, 47]
Cure rate genotype 1 SOC	71	66–75	652–646	Cure rates for boceprevir and telaprevir [14]
Cure rate genotype 2/3 SOC	81	76–86	650–648	5 % on either side of baseline scenario

Variable	Baseline scenario	Input range	Output range (baseline: $3142.26)	Input range References/comments
Model 3				
Standard of Care				
SOC genotype 1 cure rate in naïve (ribavirin + interferon + PI)	71 %	66–75 %	$3635.87–$2775.43	Cure rates for boceprevir and telaprevir [14]
SOC genotype 1 length of therapy (ribavirin + interferon + PI)	48 weeks	24 weeks	$732.86	[41]
SOC genotype 1 proportion using Pegasys	50 %	0–100 %	$2220.11–$4064.20	Theoretical range
SOC genotype 1 proportion using boceprevir	50 %	0–100 %	$2755.10–$3656.21	Theoretical range
SOC genotype 1 proportion using telaprevir	50 %	0–100 %	$3656.21–$2755.10	Theoretical range
SOC genotype 2/3 cure rate (ribavirin + interferon)	81 %	76–86 %	$3315.43–$2959.13	5 % on either side of baseline scenario
SOC genotype 2/3 length of therapy (ribavirin + interferon)	24 weeks	12–48 weeks	$2880.16–$3666.15	[48]
SOC genotype 2/3 proportion using Pegasys	50 %	0–100 %	$2971.95–$3312.37	Theoretical range
All Oral				
Proposed all oral genotype 1 cure rate in naiive (sofosbuvir + ledipasvir)	97 %	95.4–97.7 %	$2979.75–$3203.16	[44]
Proposed all oral genotype 1 length of therapy (sofosbuvir + ledipasvir)	12 weeks	8–24 weeks	$4620.24–($1292.14)	[44]
Proposed all oral genotype 1 treatment cost (sofosbuvir + ledipasvir)	85,000	106,250–127,500	$2033.58–$925.01	[35]
All oral genotype 2 cure rate in naiive (sofosbuvir + ribavirin)	95 %	90–100 %	$3001.47–$3224.89	5 % on either side of baseline scenario
All oral genotype 2 length of therapy (sofosbuvir + ribavirin)	12 weeks	16 weeks	$2798.80	[45]
All oral genotype 3 cure rate in naiive (sofosbuvir + ribavirin)	93 %	88–98 %	$3062.48–$3214.03	5 % on either side of baseline scenario
All oral genotype 3 length of therapy (sofosbuvir + ribavirin)	24 weeks	N/A	N/A	N/A

#### Disease endpoints

There were four mutually exclusive chronic HCV disease endpoints incorporated into this model: (1) cured, (2) not cured and no cirrhosis, (3) not cured with cirrhosis and no end-stage liver disease (ESLD), and (4) not cured with cirrhosis progressing to ESLD. The flow diagrams incorporating cure rates and disease endpoints for each therapeutic scenario are shown in Fig. 2b–d.

#### Cost assumptions

Total costs were calculated by summing costs for one course of treatment for HCV and 14 years of subsequent, all-cause medical care across the four mutually exclusive disease endpoints. To estimate treatment costs, we assumed that all patients were treated in 2013 and were prescribed and adhered to a full course of therapy. The treatment regimens that formed the basis upon which treatment costs were calculated can been found in Table 1. We used wholesale acquisition costs to estimate the cost of medications that were FDA approved. For the one therapy that was not FDA approved, genotype 1 all oral fixed dose combination therapy consisting of sofosbuvir and ledipasvir, we estimated its cost based on market insights from BioMedTracker^SM^ [35].

We chose to examine medical treatment for 14 years subsequent to treatment because that is the estimated length of time it takes for progression through cirrhosis to ESLD (12 years) [36] and from ESLD to death (2 years) [36]. The all cause medical costs, incorporating both hospital and outpatient costs, for (1) cured and (2) not cured, no cirrhosis were based upon a study by Kaiser Permanente that examined healthcare costs after treatment by cured status [37] that we inflation adjusted to 2013 dollars [38]. We estimated the per person cost of all cause medical care in 2013 to be $7626 for cured and $12,281 for not cured without cirrhosis. For patients not cured with cirrhosis but no ESLD as well as those not cured with cirrhosis progressing to ESLD, we used estimates from an article by Gordon and colleagues [39] that calculated annual all cause medical costs for cirrhosis and ESLD and inflation adjusted them to 2013 dollars [38]. The annual all cause medical costs for treating a patient in 2013 was estimated to be $24,460 for cirrhosis and $64,497 for ESLD, respectively. For those whom we predicted would progress to ESLD, we summed the cost of treatment for compensated cirrhosis for 12 years (2013–2023) and ESLD for 2 years (2024–2026) to estimate the cost over 14 years.

Since the analysis for medical costs is forward looking over 14 years, we also inflation-adjusted costs from 2014 onward using the Centers for Medicare and Medicaid’s National Health Expenditures Average Annual Percent Change for Inflation [40]. Inflation adjustments were not available for years 2023–2026, so we averaged the inflation adjustments over 2014–2022 and used the resulting average for the years without projections. To allow flexibility, we also provided the ability to modify certain assumptions for Model 3. The SOC length of therapy is modifiable because it has been suggested that about half of those with genotype 1 disease who are treatment naïve may be eligible for a shorter course of SOC therapy [41]. Furthermore, since a payer may prefer which interferon or protease inhibitor is used; we built flexibility into the model to allow the proportion of people on each regimen to be modifiable. The default scenario assumed that 50 % used each medication when there were two options available (Table 1). Since the all oral therapy for genotype 1 disease had not been FDA-approved at the time of the analysis, the payer can also modify the per member cost for that therapy.

## Results

Using the default scenario of a payer with 100,000 adult members having an age, sex, and region distribution the same as that for the United States, we estimated that n = 1257 (1.26 %) members would be HCV antibody positive. Among those antibody positive, Model 2 estimated that n = 649 (52 %) would have chronic disease that remained uncured. This group combines those who are in the Baby Boomer generation and have not previously been diagnosed, those who have been diagnosed and have not been treated or are awaiting new therapy, and persons who were previously treated but remained uncured. Together they represent the treatment and medical burden to the payer over the next 14 years (Fig. 2).

**Fig. 3:**
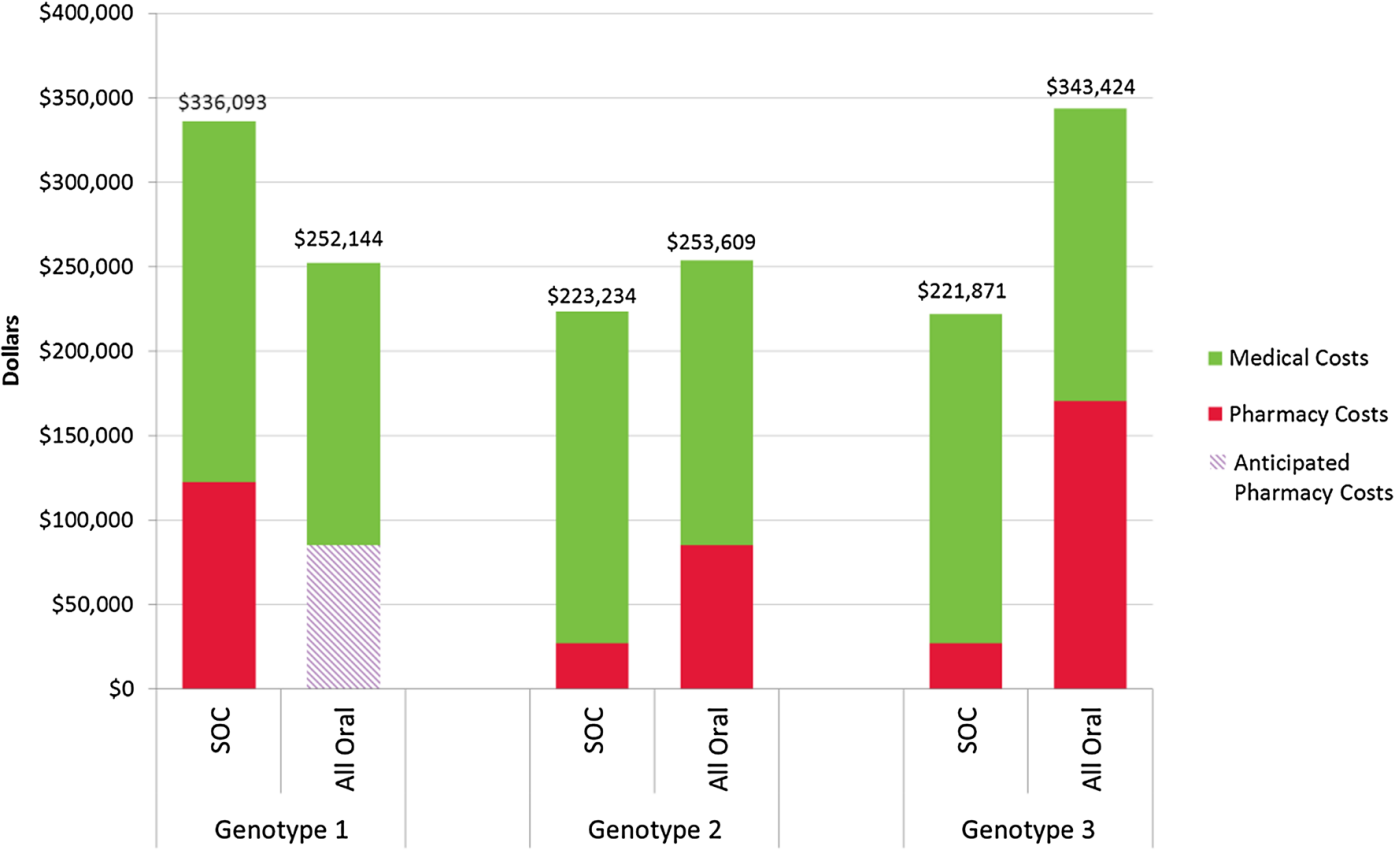
Average per patient costs over 14 years, Standard of Care (SOC) vs. All oral by Genotype. “SOC” = Standard of Care = interferon-based treatment used prior to November, 2013. “All Oral” = All Oral Therapy = various combinations of sofosbuvir, ledipasvir and ribavirin

In Model 3, we first contrasted the treatment and medical cost burden over 14 years between all oral therapy and SOC by genotype. For genotype 1 disease, the per member HCV treatment cost was 31 % lower for all oral therapy compared to SOC ($85,000 vs. $122,401) (Table 2). In addition, the cure rate for all oral therapy compared to SOC was estimated to be 37 % higher (97 vs. 71 %), which led to medical cost savings of approximately $3325 per patient per year for genotype 1. The overall per member net decrease in costs (pharmacy + medical) between all oral and SOC for genotype 1 was $5996 per patient per year. For genotype 2 disease, the per member HCV treatment cost was 213 % higher for all oral therapy compared to SOC ($85,084 vs. $27,206), however the cure rate for all oral treatment was also 17 % higher (95 vs. 81 %). With a medical cost savings of only $1965 per patient per year between all oral and SOC, there was an overall net increase in costs of $2170 per patient per year for genotype 2 (Fig. 3). For those with genotype 3 disease, the per member HCV treatment cost was 525 % higher for all oral therapy vs. SOC ($170,167 vs. $27,206). Even with a cure rate for all oral therapy that was 15 % higher (93 vs. 81 %) there remained a net increase in costs between all oral and SOC of $8682 per patient per year for genotype 3 (Fig. 3).

**Fig. 4:**
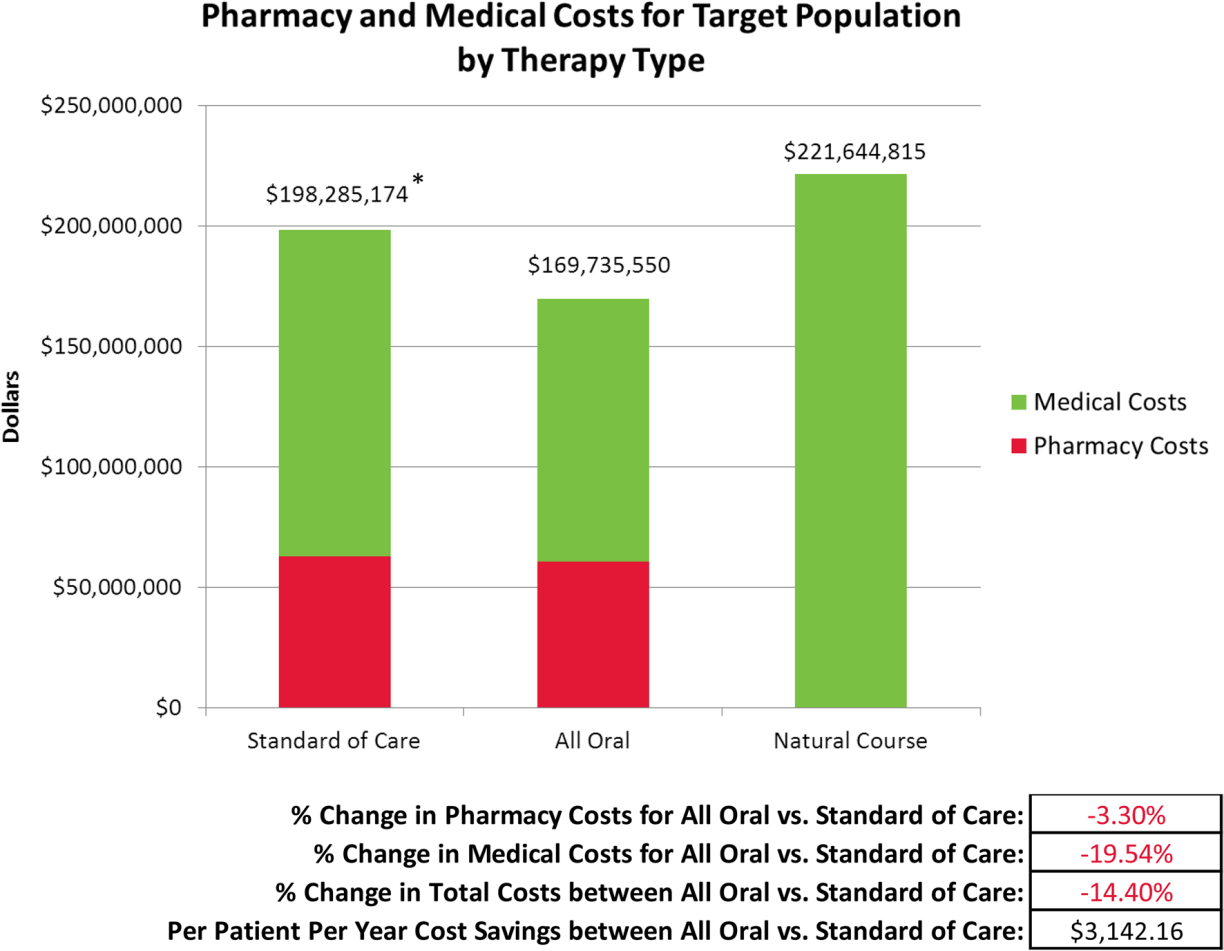
Total pharmacy and medical costs over 14 years by therapy type. “SOC” = Standard of Care = interferon-based treatments used prior to November, 2013. “All Oral” = All Oral Therapy = various combinations of sofosbuvir, ledipasvir and ribavirin. “Natural Course” = Natural Course of Disease Progression = no treatment

**Table 2:** Pharmacy cost assumptions within each therapeutic scenario by genotype

Therapeutic scenario	Genotype	Drug	Daily dosage	WAC 1 week	Number of weeks	Regimen cost per person	Regimen references
Standard of Care (SOC)^d^	1	Peginterferon alpha-2a or peginterferon alpha-2b	180^a,b^ or 120 mcg/0.5 ml^a,b^	$1441.60^e^ or $705.16^e^	48	$122,401.44	[10, 14]
		Ribavirin	1200 mg^b,c^	$90.30	48		
		Boceprevir or telaprevir	2400 or 2250 mg	$1521.24^e^ or $5512.92^e^	44 or 12		
	2/3	Peginterferon alpha-2a or peginterferon alpha-2b	180^a,b^ or 120 mcg/0.5 ml^a,b^	$1441.60^e^ or $705.16^e^	24	$27,205.92	[10]
		Ribavirin	800 mg	$60.20	24		
All oral	1	Sofosbuvir	400 mg	$7083.33	12	$85,000	[44]
		Ledipasvir	90 mg				
	2	Sofosbuvir	400 mg	$7000	12	$85,083.60	[45]
		Ribavirin	1200 mg^b,c^	$90.30	12		
	3	Sofosbuvir	400 mg	$7000	24	$170,167.20	[45]
		Ribavirin	1200 mg^b,c^	$90.30	24		

*WAC* wholesale acquisition cost

^a^Weekly administration

^b^Weight-based regimens were calculated based on the average weight of an adult, combining males and females, in the United States (181 lbs) [46]

^c^Ribavirin cost was calculated based on the median WAC cost for a 200 mg capsule

^d^Defined as interferon-based therapy that was considered SOC up to November 2013

^e^When there was a choice between two drugs, half of the group was assigned to each drug

After taking into account the prevalence of each genotype for the n = 649 members from Model 2, there was an overall net cost savings between all oral vs. SOC therapeutic scenario totaling $3142 per patient per year (Fig. 4). The cost savings resulted from a 3 % reduction in pharmacy costs and a 20 % reduction in medical costs for all oral therapy vs. SOC overall. Of note, the natural course of disease was the most costly method of all three therapeutic scenarios (Fig. 4).

We conducted a sensitivity analysis for all three models by showing the resulting change in the output of the model based on the range of values for one input (Table 1). As can be seen in Table 1, Model 1 was most affected by the age distribution of the population in comparison to other demographics. Changes to inputs for Model 2 did not result in much variability in the number of chronic uncured members with HCV that were expected to receive treatment in the next 14 years. For Model 3, the three variables that had the most effect on the per patient per year costs were: (a) length of therapy for genotype 1 SOC, (b) length of therapy for anticipated genotype 1 all oral therapy, and (c) cost of the anticipated all oral genotype 1 regimen. Increasing the length of therapy for the anticipated all oral therapy for genotype 1–24 weeks resulted in a cost increase for all oral therapy vs. SOC. However, this scenario is very unlikely to occur in a group where a large proportion is treatment naïve, requiring a shorter treatment length.

**Fig. 5:**
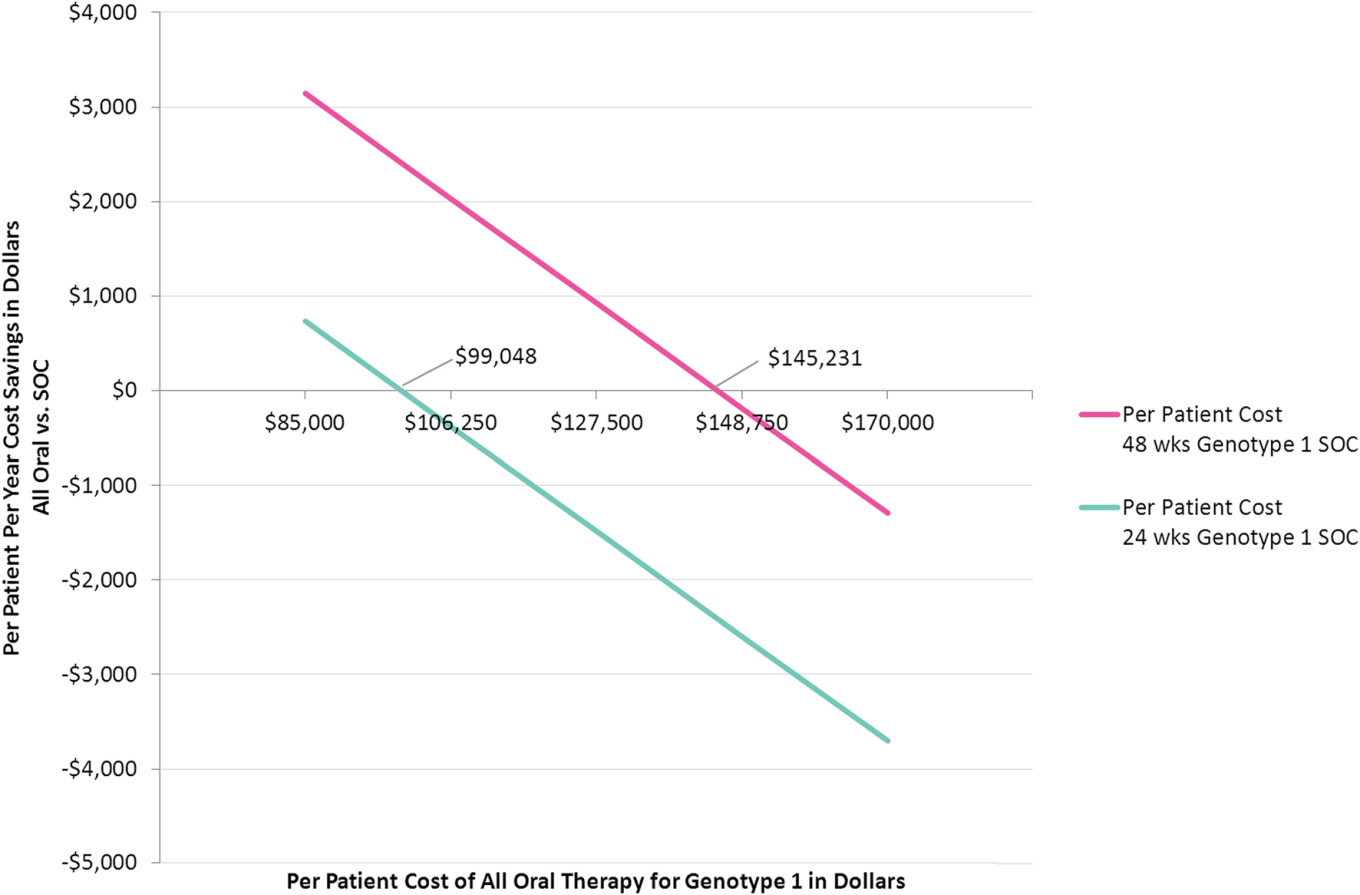
Per patient per year cost savings: All Oral vs. SOC by cost and length of therapy for genotype 1 All Oral Therapy. “SOC” = Standard of Care = interferon-based treatments used prior to November, 2013. “All Oral” = All Oral Therapy = various combinations of sofosbuvir, ledipasvir and ribavirin

We also performed a break even sensitivity analysis by allowing the projected price point for genotype 1 all oral therapy to vary ($85,000–$170,000) as well as the length of therapy for genotype 1 SOC (24 vs. 48 weeks) (Fig. 5). The results of the sensitivity analysis show that the cost for genotype 1 all oral therapy could not exceed approximately $99,000 per member in order for the all-oral therapeutic scenario to avoid being more costly than SOC when genotype 1 SOC length of therapy was 24 weeks. At the length of therapy threshold of 48 weeks for genotype 1 SOC, the cost of genotype 1 all oral therapy could not exceed approximately $145,000 per member in order for the all oral therapeutic scenario to avoid being more costly than SOC (Fig. 5).

## Discussion

This set of integrated economics allows payors to estimate the burden of uncured chronic HCV in their population as well as contrast the pharmacy and medical costs for 14 years between three therapeutic scenarios. In our study (Fig. 3) the cost savings for all oral therapy over SOC comes from the superior cure rate of all oral therapy to treat genotype 1 disease, which led to a medical cost reduction. Since the majority of the population will be infected with genotype 1, this cost savings was enough to lead to a net cost savings when comparing all oral therapy to SOC or natural course. Moreover, the results show that if a payer allows all of the uncured chronic HCV members to utilize all oral therapy upon diagnosis, there would likely be cost savings for the payer at the end of 14 years.

### Comparison to other published studies

We found two other published studies examining the benefit of all-oral therapy vs. interferon-based SOC. In 2013, Hagan and colleagues published findings of a Markov model they built to examine a middle-aged cohort as it progressed through HCV disease to death. The outcomes of the model were willingness-to-pay threshold, incremental cost-effectiveness ratio, and quality-adjusted life years. Across these outcomes, the all-oral therapy for genotype 1 disease was most cost effective. However, there were also scenarios where all oral therapy, as compared to an interferon-based regimen, was cost-effective for genotypes 2 and 3 disease. The authors noted that variables such as the provider’s willingness to pay threshold as well as the cost of all oral therapy were two key factors in determining cost effectiveness [20].

A recently published article by Younossi and colleagues compared interferon-based and all oral therapy among genotype 1 HCV using both clinical and monetary outcomes [42]. The unique feature of this study was the additional analysis examining a staged (i.e. severity of disease) approach vs. a treat everyone approach. The authors concluded that all-oral therapy was more cost effective compared to interferon-based therapy. Furthermore, they found that treating everyone could also be cost effective as it resulted in the fewest number of people progressing to end-stage liver disease [42]. While approaches and outcomes across these studies and the current study differed, the results point to the same conclusion, which is that all oral therapy can be cost effective when compared to interferon-based treatment for HCV.

### Limitations

This study had several limitations. First, the models only apply to adults with HCV as Model 1 only encompasses persons 18 years of age and older. Second, the models are not reflective of HCV prevalence, cure rates, and medical expenditures for special populations, such as those co-infected with human immunodeficiency virus (HIV) or a prison population. Rather, they represent a commercially insured population.

In Model 1, we aged the population to project age-specific HCV prevalence from 2005 to 2007 to be applicable to the years 2012–2013. This methodology is limited in that it does not take into account entries (new infections) or exits (deaths) amongst the population over this time. If the ratio of new infections to deaths changed from 2005 to 2007 to the 2012–2013 period, our Model 1 would lead to an over- or underestimate of HCV prevalence affecting cost outcomes for Model 3. However, this limitation is not expected to affect our main outcome; the per patient per year cost comparison between all oral and SOC therapy.

For Model 2, the estimated prevalence of diagnosed chronic HCV is based on data from a managed care population and may not be generalizable to the general population in the United States. Evidence shows that in contrast to a managed care population, only about one quarter of those in the general population with HCV have been diagnosed [43].

With respect to Model 3, all of those in the uncured population with chronic HCV are assumed to be tested and treated for HCV within the first year. Thus, pharmacy costs may be underestimated, as they are not inflation adjusted. In addition, the proportion of the uncured population with chronic HCV that undergoes treatment and progresses to end-stage liver disease may be underestimated, as the proportion developing cirrhosis does not account for the increased likelihood of those with disease that is more advanced or prior treatment failure not being cured. However, many asymptomatic will be diagnosed per the United States Preventive Services Task Force (USPSTF) recommendations and almost all of those that will be treated in our Model 3 will be treatment naïve (96 %). Thus, the impact of this limitation is expected to be minimal.

Furthermore, the pharmacy costs only take into account one course of therapy, which may lead to an underestimate of total pharmacy costs for the SOC and all oral therapeutic models depending on the need for re-treatment. In addition, the cost estimate for genotype 1 all oral therapy is only a projection, because the therapy had not been approved by the time the estimates were developed [26]. To mitigate the uncertainty of this projection, we included the break even sensitivity analysis that provides a range of costs that might be expected for the per person cost of genotype 1 all oral therapy with the resulting impact to the outcome of Model 3. In addition the regimen and accompanying cure rate for genotype 1 all oral therapy is only given as an example because the fixed dose combination of sofosbuvir and ledipasvir had not yet been FDA-approved, and the cure rate is based on phase III clinical trials for this combination [44].

The medical and pharmacy costs do not take into account liver transplantation or treatment for hepatocellular carcinoma as the prevalence of these disease endpoints were too rare to contribute meaningfully to the cost estimates for the default scenario. The impact of such disease endpoints, if included with a larger population, would likely lead to a greater cost savings for an all oral therapy therapeutic scenario compared to SOC, since the cure rate for the all oral therapy is higher. Model 3 also does not account for the cost to treat side effects. Since all oral therapy is predicted to have fewer side effects than SOC [20, 21], Model 3 is likely conservative with respect to the cost-savings of the all oral therapeutic scenario compared to SOC. In addition, Model 3 does not address the period from diagnosis of chronic HCV until the development of cirrhosis for those who will develop cirrhosis, with or without ESLD. Since many of the newly diagnosed will result from birth cohort screening, the latency period before cirrhosis develops is anticipated to be small as disease acquisition likely occurred 20–30 years ago [3]. Finally, the costs modeled are representative of what may be incurred over 14 years. Thus, results may not be applicable to costs or savings incurred in the short term.

## Conclusion

As more people are diagnosed with HCV in the coming months and years, payers will need to project the disease burden of HCV in their member groups and weigh the economic benefit of providing new all oral therapy vs. SOC. Our models take into account a population-based perspective factoring in not only the cost of therapy, but also the resulting medical costs over 14 years, while accounting for the prevalence of each genotype within the member group. Based on our results, all oral therapy can result in cost savings over time when treating patients with HCV. As new all oral therapies for HCV are brought to market and the price for these newly FDA approved medications are assigned, the cost benefit for all oral therapy vs. SOC will continue to evolve. While our model shows that the new therapies do indeed create medical cost savings, there remains controversy over how can the short-term drug costs be afforded by payers who typically do not have consistent membership over time. Thus, an insurer will be pay for a patient’s cure while another insurer or Medicare, will be the ones to enjoy the downstream financial benefit years later. More consideration should be given to how these drugs are financed in the United States, so that those paying for the therapies can be assured that their investment will generate downstream financial benefits.
